# Inclusion of pregnant and lactating women in TB treatment and vaccine trials

**DOI:** 10.5588/ijtldopen.26.0107

**Published:** 2026-09-15

**Authors:** S-A. Meehan, A.C. Hesseling, L. Viljoen, L.M. Cranmer, S. Verkuijl, K. Viney, M. Maktabi, M. Isaacs, Y. Hirsch-Moverman

**Affiliations:** 1 Desmond Tutu TB Centre, Department of Paediatrics and Child Health, Faculty of Medicine and Health Sciences, Stellenbosch University, South Africa;; 2 Emory University, Atlanta, GA, USA;; 3 Children’s Healthcare of Atlanta, Atlanta, GA, USA;; 4 Department of HIV, Tuberculosis, Hepatitis and Sexually Transmitted Infections, World Health Organization, Geneva, Switzerland;; 5 Department of Epidemiology at Mailman School of Public Health, Columbia University, New York, NY, USA;; 6 ICAP at Columbia University, Mailman School of Public Health, Columbia University, New York, NY, USA.

**Keywords:** pregnancy, tuberculosis, treatment regimen, prevention, vaccines

## Abstract

**BACKGROUND:**

Historically, pregnant and lactating women (PLW) have been excluded from clinical trials for TB, thereby denying them access to novel treatment and prevention regimens. We aimed to develop an inventory of TB therapeutic and vaccine trials and determine the barriers and facilitators to the inclusion of PLW in TB clinical trials.

**METHODS:**

Using a mixed-methods approach, we conducted: a review of recent, current, and planned trials’ inclusion and exclusion practices, and in-depth interviews with trialists and sponsors.

**RESULTS:**

Of 40 TB therapeutic trials, 7/40 (18%) included PLW; 3/40 (8%) excluded PLW at enrolment but allowed women to remain on study treatment if they became pregnant. None of 12 vaccine trials included PLW but 6/12 (50%) included PLW in safety and/or immunogenicity assessments during study follow-up. Eighteen participants completed in-depth interviews. We identified four broad themes: shifting the narrative, differentiated risks, perceived concerns of PLW, and strategies for inclusion.

**CONCLUSIONS:**

With limited inclusion of PLW in TB therapeutic trials and no inclusion in vaccine trials at the time of enrolment, there was consensus that clear guidance is required for trialists, funders, and sponsors on strategies for inclusion of PLW, thus ensuring equitable access to novel TB prevention and treatment regimens.

Compared to the risk of TB in non-pregnant women, the risk of TB is one and a half times higher during pregnancy and twice as high during the postpartum period.^1–3^ More than 230,000 pregnant women and almost 100,000 postpartum women develop TB annually.^4^ Pregnant women with TB and TB/HIV are at high risk of dying compared to non-pregnant women.^5^ TB during pregnancy carries a high risk of adverse pregnancy outcomes including miscarriage, preterm birth, and low birth weight.^6,7^ Having an unfavourable TB treatment outcome is associated with adverse pregnancy outcomes.^8^ Including pregnant and lactating women (PLW) in clinical trials for novel TB therapeutics for prevention, treatment, and vaccine trials is essential to optimise health outcomes for this population. PLW have been routinely excluded from TB clinical trials. As a result, policy makers are unable to make recommendations that can be extrapolated to PLW. PLW are thus denied equal access to novel TB treatment and prevention options. In 2013, a panel of experts published a consensus statement arguing for the accelerated inclusion of PLW in TB clinical trials.^9^ In 2023, communities affected by TB called for the active inclusion of PLW in TB trials.^10^

Regulatory pathways define the requirements for including PLW in clinical trials, typically mandating reproductive toxicity data, prior safety evidence, and ethics and regulatory approvals. These frameworks have historically favoured a cautious, stepwise approach, delaying the generation of pregnancy-specific evidence. When PLW are included, enhanced pharmacovigilance is required to monitor maternal, foetal, and infant outcomes.^11,12^ Stakeholders, including health care workers, ethics committees, funders, and trial sponsors, often regard standard TB treatment regimens as sufficiently safe for PLW, fostering a risk-averse approach that prioritises avoidance of trial-related harms over evidence generation. Coupled with assumptions that PLW are unwilling to participate in trials of novel TB therapeutics,^13–15^ this has contributed to limited momentum to evaluate new regimens.

To support the WHO-led consensus process (2024–2025) on the earlier and optimal inclusion of PLW in TB clinical trials,^16^ and to address gaps in published data that quantify inclusion and exclusion criteria across TB therapeutic and vaccine studies, we aimed to develop a global inventory of TB therapeutic and vaccine trials and identify key barriers and facilitators to the inclusion of PLW in these trials.

## METHODS

Using a mixed-methods approach, we conducted i) a review of inclusion and exclusion practices for PLW in recent, current, and planned TB therapeutic and vaccine trials and ii) in-depth interviews (IDIs) to determine barriers and facilitators to inclusion of PLW in TB drug and vaccine trials.

### Review of TB trials

Using a pragmatic approach, we developed a systematic inventory of recent (≤10 years as of 2024), current, and planned TB prevention, treatment, and vaccine trials based on a review of clinical trial registration sites: ‘ClinicalTrials.gov’ and the ‘International Clinical Trials Registry Platform’. We consulted global trial networks, investigators, industry sponsors, and funders to ensure a comprehensive inventory. In consultation with the WHO, we included Phase III and IV trials for TB therapeutics and Phase II and III trials for TB vaccines. We created a database organised into 1) treatment of TB (drug-susceptible and drug-resistant TB), 2) TB preventive treatment (TPT), and 3) TB vaccines. Using published trial protocols, we indicated if the trial included PLW at enrolment or excluded them at enrolment but allowed those who became pregnant on study to remain in the trial and receive study product.

### In-depth interviews

To explore reasons for inclusion/exclusion of PLW, we developed a semi-structured interview guide, in consultation with the WHO secretariat. The guide included information on attitudes about the inclusion of PLW in TB therapeutic and vaccine trials during pregnancy and lactation. We purposively enrolled trial sponsors and clinical trialists, utilising the information collected during the review to identify potential participants, ensuring diversity across participant category, trial type, phase, PLW inclusion/exclusion criteria, and geographical location. Participants were interviewed online at their convenience. IDIs were conducted in English between November 2024 and January 2025 by experienced qualitative TB researchers.

### Data analysis

Trial data were summarised using descriptive statistics, including numbers and proportions. Rapid qualitative analysis of IDIs was performed using the rigorous and accelerated data reduction (RADaR) technique.^17^ We summarised interview transcripts and developed a template summary table in Excel. A deductive approach was used as the first line of analysis to develop themes. An inductive approach was then utilised to identify emergent sub-themes and contextualise findings.

### Ethical statement

The protocol was approved by the Columbia University Irving Medical Center Institutional Review Board (Ref AAAV4735) and the Stellenbosch University Health Research Ethics Committee (Ref N24/09/108). All participants provided written informed consent.

## RESULTS

### Inventory of TB Trials

We identified 40 therapeutic clinical trials; 29 treatment trials, 11 TPT trials, and 12 TB vaccine trials. Overall, 18/52 (35%) were current, 24/52 (46%) were complete, and 10/52 (19%) were planned.

Of 29 treatment trials, 3/29 (10%) included PLW, and 2/29 (7%) excluded PLW at enrolment but allowed women to remain on study treatment if they became pregnant. Of 11 TPT trials, 3/11 (27%) included PLW, 1/11 (9%) included lactating women, and 1/11 (9%) excluded PLW at enrolment but allowed women to remain on study treatment if they became pregnant (Supplementary Data Table S1).

Of 12 vaccine trials, none included PLW but 6/12 (50%) included PLW in safety and/or immunogenicity assessments during study follow-up (Supplementary Data Table S2).

### In-depth interviews

Of 31 potential participants, 11 did not respond to the email invitation, 2 refused due to time limitations, and 18 enrolled (16 clinical trialists and 2 sponsors). The majority (13/18; 72%) identified as women. The median years’ experience working in TB was 15. Two thirds of participants had experience in both low- and middle-income countries (LMICs) and high-income countries. Most (13/18; 72%) participants specialised in TB therapeutic research; seven of these participants were also able to comment on vaccine research. Five participants specialised in vaccine research; two of which could also comment on therapeutic research.

We identified four broad themes. Illustrative quotations for each theme are presented in Supplementary Data Table S3 (therapeutics) and Supplementary Data Table S4 (vaccines).

### Shifting the narrative

Participants agreed that TB research among PLW has increased in recent years, but significant gaps remain, including the need to progressively shift the narrative from ‘automatic exclusion’ to ‘automatic inclusion’ with individual exclusion only if the relevant risks outweigh the benefits.

Participants explained that TB trial design is rooted in historic practices where TB researchers would routinely exclude PLW due to safety risks and to avoid potential regulatory challenges or litigation risks. Participants noted that they often found it easier to replicate exclusion criteria from previous study protocols, though some noted that they tried to change this automatic exclusion. Strict sponsor requirements and influence were cited by trialists as potential barriers to PLW inclusion. Many sponsors were seen as unsupportive of enrolment of pregnant women, due to the lack of prior drug approval for use in pregnancy. As a result, researchers adhered to this requirement, which reinforced the exclusion of PLW. Obtaining clinical trial insurance (CTI) for a study to include PLW could delay the study launch and was noted as a barrier to inclusion. Some participants noted that there were countries with experience in obtaining CTI for vulnerable populations, which facilitated the inclusion of PLW in TB trials. Participants reported a lack of clear, pragmatic guidelines for including PLW in TB trials. Without such defined pathways, participants reported that they were unsure on how to proceed with trial enrolment and therefore found it easier to exclude PLW.

Participants reported that PLW received better standard of care when they participated in trials, specifically in LMICs, which was thought to ultimately contribute to helping improve pregnancy outcomes in these settings. Participants further noted that pregnant women who participate in pregnancy-focused clinical trials were seen to especially benefit since those trials would be conducted under close regulatory scrutiny and would usually provide additional safety assessments, for example, ultrasound monitoring and other procedures that would not usually be available. Several participants noted that allowing lactating women to take part in trials could lead to closer monitoring and improved outcomes for mothers and their infants. They also noted that, if drug levels in breast milk were therapeutic, trial participation could offer additional protective benefits for infants.

### Differentiated risks

Participants stated that PLW remain a high-risk population, which had been the major barrier to their automatic inclusion in clinical trials to date. They highlighted the importance of separating the pregnancy and lactation periods, as the risks to mother and child are quite different. Some participants noted that in trials that specifically focus on pregnancy outcomes, excluding lactating women is logical. Implementing trials during the lactation period is simpler compared to during pregnancy, as lactation involves far fewer physiologic changes, making drug behaviour more predictable and reducing safety uncertainties. Participants suggested that for prevention trials, participation of PLW could be deferred until they are no longer lactating.

For pregnancy, trimester was reported as an important consideration, with the second and third trimesters regarded as being acceptable times to include pregnant women in therapeutic trials. Several participants noted that it was important to consider including women in the first trimester because TB can occur during any time of the pregnancy; however, it was noted that safety data are required before enrolling women in their first trimester of pregnancy.

Assessment of drug safety is crucial when planning TB therapeutic trials. Participants felt that it was important to screen for potential risks before enrolling pregnant women and to ensure that the drug is not harmful for the pregnancy. Additionally, the importance of appropriate dosing was raised. Participants noted that exclusion criteria for lactating women should be based on the safety of the drug and potential drug transfer/toxicity to the breastfed baby based on animal and human data. Participants also highlighted the need to consider safety during the lactation period by regularly monitoring infants.

The context where the research is conducted was identified as a potential risk factor for safety and monitoring. It was reported that some countries have stronger research infrastructure and more experience, which may better support the inclusion of PLW in clinical trials.

Participants suggested weighing the risk–benefit for each trial as the severity of TB sometimes drives inclusion/exclusion decisions. The more severe the form of TB, the more willing investigators may be to take potential risks because the disease itself is associated with substantial risk of morbidity and mortality to PLW and their infants.

Participants agreed that the risk–benefit ratio for TB prevention and treatment trials were also different. Investigators may be willing to accept more risk when studying therapeutics for the treatment of TB disease, especially in severe forms such as TB meningitis, or drug-resistant TB, where treatment outcomes remain poorer than in drug-susceptible TB. Participants noted that a higher threshold should be met for including pregnant women in TB prevention trials; prevention was seen as an ‘option’ versus treatment, which was seen as a necessity. Some participants stated that including lactating women in prevention trials could be seen as part of postpartum care. Programmatically, this is an ideal time, as women interact with health care systems regularly during the postpartum period, including for infant wellness and vaccination. In addition, the risk for TB is higher during the postpartum period than during pregnancy.^1^

Participants commented on the specific risks related to TB vaccine trials: i) the type of vaccine, where there was agreement that live vaccines should not be used in pregnancy but there was openness to the use of other vaccine types, ii) timing of vaccination where views varied regarding vaccinating during pregnancy or lactation, with some participants considering the time to vaccine efficacy while others were noting time to immunogenicity, and iii) weighing the risks and benefits, for example, while vaccinating PLW against TB presents an opportunity to prevent TB, especially in high-burden settings, this needs to be weighed against potential side effects for the pregnant woman as well as for the pregnancy.

### Perceived concerns of PLW

Most participants expected that pregnant women would be most concerned about added risk to the foetus and themselves and may turn to their health providers for more information. Participants felt that pregnant women could be concerned that TB drugs may cause abnormalities to the baby or may lead to early termination of the pregnancy. Lactating women would be most concerned about the presence of drug in breastmilk and the risk this poses to their infants. For novel TB vaccines, pregnant women could be concerned about the risk of potential congenital anomalies, preterm birth, and if being vaccinated would affect the effect of foetal growth. Participants noted that women may find the time commitment for trial participation challenging; the additional burden of clinical trial procedures at a time when they may not feel well due to having TB while having some effects from the pregnancy or balancing trial procedures with the demands of caring for a newborn. These challenges could potentially lead to either non-participation or early withdrawal from the trial.

### Strategies for inclusion of PLW in TB clinical trials

Participants stated that it is important to learn from trials where pregnant women were initially excluded but allowed to remain on the study drug if they became pregnant. Drawing on these lessons and their experience, participants identified several important strategies in progressing toward inclusion of PLW in trials (see Figure).

**Figure.**
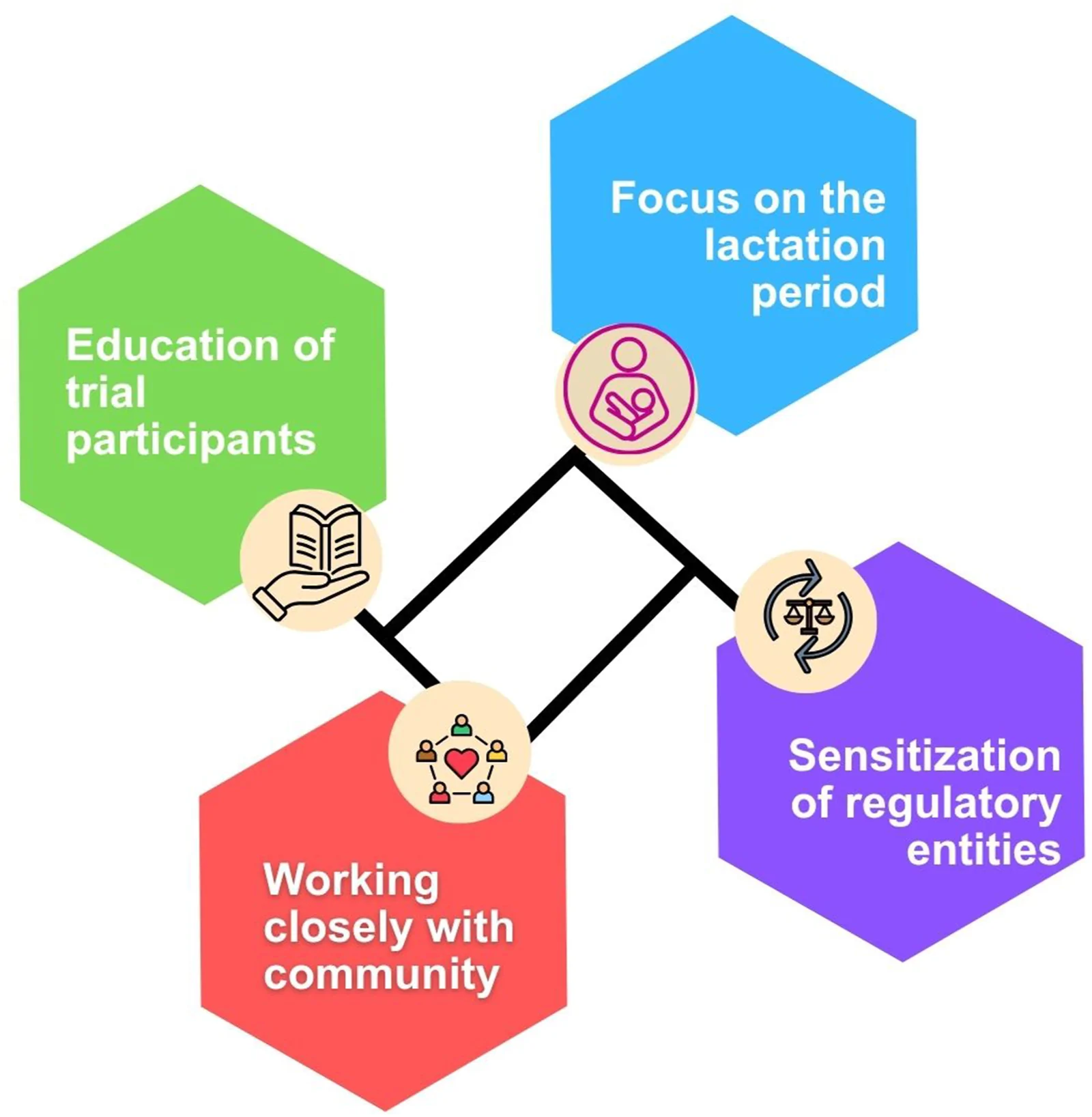
Strategies for inclusion of pregnant and postpartum women in TB clinical trials.

Strategy 1 – Working closely with the community: participants envisioned that the community could play a significant role in creating awareness and educating communities around the importance of including PLW in TB clinical trials. They mentioned community-based advocacy and noted a move in recent years from focusing on the protection of pregnant women from ‘dangerous’ drugs to demanding that they are included in trials so that they can benefit from advances in TB treatments.

Strategy 2 – Educating trial participants about potential benefits and risks of TB trial participation: participants noted the need for trialists and researchers to educate PLW and their families about the potential benefits and risks of trial participation. This education should include an effective informed consent process that is honest about procedures, to build trust between the women, their families, and researchers. In addition, pregnant women’s concerns could be alleviated if it was clearly conveyed that trial participation would result in closer monitoring of their TB treatment and pregnancy outcomes.

Strategy 3 – Sensitisation of regulatory entities: several participants noted the need for sensitisation and awareness building of regulatory entities. Education should include details and motivation of the movement toward inclusion of PLW in research generally, as well as specifically in TB research.

Strategy 4 – Focus on the lactation period: lactation is a good time to enrol women in TB trials since the postpartum period is a period of risk for developing TB, and women are already engaged with health care systems for early infant visits, and there is reduced risk compared to enrolling during pregnancy, with a potential risk to the in utero infant. Participants noted the need to develop strategies that will facilitate inclusion, for example, advising lactating women on strategies for timing breastfeeding to minimise drug exposure in breastmilk.

## DISCUSSION

The exclusion of priority populations,^13^ particularly PLW^11^ in TB trials, represents a significant and unacceptable gap in TB research. PLW deserve equitable access to effective and safe TB prevention and treatment.^18^ Their exclusion not only denies them the benefits of the latest advancements in TB prevention and treatment but also perpetuates a cycle of inadequate evidence, delayed access, and suboptimal clinical guidance, tailored to their needs. A TB pregnancy and lactation toolkit will be a vital resource for providing practical and clinical guidance on the inclusion of PLW in TB clinical trials.

PLW have historically been excluded from TB clinical trials. Our findings support this; only 18% of Phase III and IV therapeutic trials and no Phase II and III vaccine trials conducted since 2014, or those ongoing or planned, included PLW at enrolment. While there is growing awareness and increasing advocacy around the need to include this priority population in TB clinical trials, progress remains inadequate. The WHO global consensus process^16^ to ensure the earlier and optimal inclusion of PLW in TB trials is therefore timely and pertinent.

The notion of ‘automatic exclusion’ of PLW is a critical barrier to their equitable inclusion in clinical research,^18^ driven by numerous perceived barriers and assumptions. Strict sponsor requirements, lack of clear guidelines, anticipated delays and rejection from ethics committees, and difficulties in receiving CTI were barriers noted that can be mostly overcome if there is a willingness to address them. It has been suggested that ethical issues can be addressed by careful consideration of the trial design and inclusion of content experts, regulatory specialists, and community participation to ensure appropriate trial design and implementation.^13^ Consensus statements from an international panel of experts called for industry and trial sponsors to complete reproductive toxicity studies early in drug development, before beginning Phase III trials, to generate data to inform decisions about the appropriate inclusion of pregnant women in subsequent clinical trials.^11,16^ Regulatory authorities can have a default inclusion policy, whereby researchers would need to justify the exclusion of PLW from their research instead of having to justify their inclusion.^19^

Including all PLW in TB clinical trials is not appropriate; inclusion must balance scientific necessity, ethics, and safety. Consideration of differentiated risks was a major finding; participants emphasised differentiated risk by trial type (treatment, prevention, vaccine) and population (pregnant vs. lactating). Pregnancy and lactation present distinct risks and considerations for clinical trial participation. Trials conducted during lactation are generally less complex to implement than those during pregnancy. For prevention trials, some women may prefer to defer participation until after completing breastfeeding. Higher risk may be more acceptable when studying therapeutics, especially for severe TB, than for prevention. TB vaccination offers prevention benefits in high-burden settings but requires careful assessment of maternal and foetal safety.

HIV further complicates inclusion due to added safety considerations. Co-morbidities such as HIV, diabetes, and undernutrition can confound safety signals and challenges in attributing adverse outcomes to investigational products versus underlying conditions. As a result, even in the absence of explicit exclusion criteria, the more complex risk profiles of PLW can lead to their exclusion from clinical trials.^20–22^

Most participants perceived that pregnant women fear risks to the foetus and themselves while lactating women worry about drug presence in breastmilk and the risk to the growing infant. Consistent with this, literature shows that concern for the foetus or baby strongly influences trial participation decisions.^23,24^ Yet, in a single centre study in Ethiopia, overall willingness to receive new TB vaccines during pregnancy and lactation was high >90%.^25^ One important strategy identified by participants was the need for appropriate education of PLW, their families, and community on risks and benefits of TB trial participation, to support informed decision making. This could increase autonomy, which to date has been limited by their systematic exclusion from clinical trials.^26^

Beyond education on risks and benefits, participants stressed community engagement and sensitisation of regulatory entities as key strategies in progressing toward including PLW in TB trials. The WHO Call to Action similarly emphasised the importance of community engagement to end TB in PLW.^15^ Affected communities are key stakeholders and should play an integral role in study design, implementation, and dissemination.^16^ Policymakers, funders, and regulatory bodies should actively support PLW inclusion by providing resources, guidance, and training to researchers and product sponsors to address the specific needs and concerns of PLW.^27^ Advancing evidence for drug and vaccine use in PLW is a shared responsibility across researchers, ethics committees, advisory bodies, funders, developers, regulators, governments, civil society, health care providers, and the WHO.^15,28^

A major strength of this work is that we captured trials from the largest repositories of clinical trials to quantify the number of TB clinical trials that included/excluded PLW. Secondly, using a qualitative methodology not only enabled us to capture detailed responses from participants but also enabled us to capture individual experiences that reflect a broader consensus among these stakeholders. However, the study also had several limitations. It did not assess structural barriers across high- versus low-burden settings, which may affect PLW inclusion; future work should address this. The timeline for data collection was short and overlapped with holiday periods; however, a diverse purposive sample and data saturation were achieved. Only English-speaking participants were included, but this did not prevent us from obtaining a diverse sample. PLW themselves were not engaged, but ongoing work (unpublished) in high-burden settings is currently exploring their perspectives.

## CONCLUSION

This review supports global consensus efforts for the optimal inclusion of PLW in TB trials. Participants agreed PLW should no longer be automatically excluded; instead, trialists need to adopt strategies to address risks and barriers. Clear guidance for sponsors and clinical trialists is essential. This study supports a shift to default inclusion of PLW, with opt-out criteria based on risk–benefit by population group (pregnant vs. lactating) and trial type.

## Supplementary Material
