# Supplementary material for: Inclusion of pregnant and lactating women in TB treatment and vaccine trials
Source: IJTLD Open. 2026 Sep 15;3(9):574–9. doi: 10.5588/ijtldopen.26.0107 (PMC13577204; doi:10.5588/ijtldopen.26.0107)
Supplement: Supplementary file 1 [file ijtldopen26-0107_supplementarydata1.pdf]

INCLUSION OF PREGNANT AND LACTATING WOMEN IN TUBERCULOSIS TREATMENT AND VACCINE TRIALS

Sue-Ann Meehan[1*], Anneke C. Hesseling[1], Lario Viljoen[1], Lisa Marie Cranmer[2,3], Sabine Verkuijl[4], Kerri Viney[4], Mahdi Maktabi[5], Mia Isaacs[5], Yael Hirsch-Moverman[5,6]

*Corresponding author

Email address: sueannm@sun.ac.za (SM)

Affiliations:

[1]Desmond Tutu TB Centre, Department of Paediatrics and Child Health, Faculty of Medicine and Health Sciences, Stellenbosch University, South Africa

[2]Emory University, Atlanta, GA, USA

[3]Children's Healthcare of Atlanta, Atlanta, GA, USA

[4]World Health Organization, Department of HIV, Tuberculosis, Hepatitis and Sexually Transmitted Infections, Geneva, Switzerland

[5]Department of Epidemiology at Mailman School of Public Health, Columbia University, New York, NY, USA

[6]ICAP at Columbia University, Mailman School of Public Health, Columbia University, New York, NY, USA

Supplementary Table 1: Tuberculosis therapeutic and prevention trials that included pregnant and/or lactating women at enrolment or excluded them at enrolment, but allowed them to stay on trial if they became pregnant

Official Study Name	TB Type	Shortened Study Name	Description of Trial	Registry ID	Trial Phase	Planned/ Current/ Completed	Year of Study	Included/Excluded Pregnant & Lactating Women in Trial?	Allow Women who Got Pregnant to Remain During Trial? (Y/N)	Sponsor
**TB therapeutic trials for TB Disease that included pregnant and/or lactating women at enrollment (n=3)**										
Intensified TB Treatment to Reduce the Mortality of HIV-infected and Uninfected Patients With TBM: a Phase III Randomized Controlled Trial	DS-TB	INTENSE-TBM	INTENSE-TBM is randomized controlled, phase III, multicenter, 2 x 2 factorial plan superiority trial assessing the efficacy of two interventions to reduce mortality from tuberculous meningitis (TBM) in adolescents and adults with or without HIV-infection in sub-Saharan Africa: intensified TBM treatment with high-dose rifampicin and linezolid, compared to WHO standard TBM treatment.	clinicaltrials.gov: NCT04145258	3	Current	2021-2026 (estimated)	Included if <17 weeks pregnant	Unknown, information not available--maybe allow those who will complete the study before reaching the 17 week mark of pregnancy	ANRS, Emerging Infectious Diseases
Program for Rifampicin-Resistant Disease With Stratified Medicine for Tuberculosis	DR-TB	PRISM-TB	PRISM-TB is an international, multicenter, open-label, randomized, controlled, pragmatic, stratified medicine, treatment shortening, noninferiority Phase 3 clinical trial for fluoroquinolone-susceptible multidrug-resistant/rifampin-resistant pulmonary tuberculosis (FQ-S MDR/RR-TB).	clinicaltrials.gov : NCT06441006	3	Planned	2024-2029	Included	Women who are pregnant at start of trial must be at or greater than 14 weeks gestational age	University of California, San Francisco

Building Evidence for Advancing New Treatment for Rifampicin Resistant Tuberculosis (RR-TB) Comparing a Short Course of Treatment (Containing Bedaquiline, Delamanid and Linezolid) With the Current South African Standard of Care	DR-TB	BEAT-TB	BEAT Tuberculosis is a phase 3, open label, multi-centre, randomized controlled trial. The purpose of this trial is to compare the efficacy and safety of a Study Strategy consisting of 6 months of bedaquiline (BDQ), delamanid (DLM), and linezolid (LNZ), with levofloxacin (LVX) and clofazimine (CFZ) compared to the current South African Standard of Care (Control Strategy) for 9 months for the treatment of rifampicin resistant (RR-TB) Tuberculosis.	clinicaltrials.gov : NCT04062201	3	Completed	2019-2024	Included	Women of childbearing potential expected to use effective contraception throughout duration of study. Could stay on trial if became pregnant and stay on IP	Wits Health Consortium (Pty) Ltd
**TB therapeutic trials for TB Disease that excluded pregnant and/or breastfeeding women, but allowed them to stay on trial if they became pregnant (n=2)**										
Evaluating Newly Approved Drugs for Multidrug-resistant TB	DR-TB	endTB	endTB Clinical Trial a Phase III, randomized, controlled, open-label, non-inferiority, multi-country trial evaluating the efficacy and safety of five new, all-oral, shortened regimens for MDR-TB.	clinicaltrials.gov : NCT02754765	3	Completed	2016-2023	Completely excluded at first but started allowing them to remain on study IP if became pregnant mid-trial	NO, originally terminated partcipants who became pregnant but changed that mid-trial and allowed them to remain on study IP	Médecins Sans Frontières, France
Evaluating Newly Approved Drugs in Combination Regimens for Multidrug-Resistant TB With Fluoroquinolone Resistance	DR-TB	endTB-Q	endTB-Q Clinical Trial is a Phase III, randomized, controlled, open-label, non-inferiority, multi-country trial evaluating the efficacy and safety of two new, all-oral, shortened regimens for MDR-TB with fluoroquinolone resistance	clinicaltrials.gov: NCT03896685	3	Completed	2020-2024	Excluded but allowed to remain on study IP if become pregnant	NO, originally terminated partcipants who became pregnant but changed that mid-trial and allowed them to remain on study IP	Médecins Sans Frontières, France
**TB therapeutic trials for TB prevention that excluded pregnant and/or breastfeeding women at enrollment (n=4)**										

A Phase IV Randomized Double-Blind Placebo-Controlled Trial to Evaluate the Safety of Immediate (Antepartum-Initiated) Versus Deferred (Postpartum-Initiated) Isoniazid Preventive Therapy Among HIV-Infected Women in High Tuberculosis (TB) Incidence Settings		TB APPRISE	This study evaluated the safety of immediate (antepartum, or before delivery) versus deferred (postpartum, or after delivery) IPT among HIV-infected pregnant women in high TB incidence settings. HIV-infected pregnant women were randomly assigned (1:1) to one of two arms: Arm A (immediate/antepartum INH) and Arm B (deferred/postpartum INH group). Women in both arms received oral prenatal multivitamins and pyridoxine (vitamin B6) once daily from study entry through Week 40 postpartum.	clinicaltrials.gov : NCT01494038	4	Completed	2014-2017	Included	Yes	National Institute of Allergy and Infectious Diseases (NIAID)
Bedaquiline Roll-out Evidence in Contacts and People Living with HIV to Prevent TB		BREACH-TB	A Phase III, open-label, multicenter, non-inferiority trial, to compare the efficacy and safety of 4 weeks of bedaquiline (BDQ) versus a World Health Organization (WHO) -recommended regimen for preventing confirmed or probable tuberculosis disease (TBD) during 96 weeks of follow-up among people living with HIV (PLHIV) and high-risk Close Contacts (CC) of adults with Drug Susceptible (DS) or Rifampin Resistant (RR) TB.	clinicaltrials.gov : NCT06568484	3	Current	2024-2027	Included	Yes	Johns Hopkins University

Protecting Households On Exposure to Newly Diagnosed Index Multidrug-Resistant Tuberculosis Patients		PHOENIx MDR-TB	The purpose of this study is to compare the efficacy and safety of 26 weeks of delamanid (DLM) versus 26 weeks of isoniazid (INH) for preventing confirmed or probable active tuberculosis (TB) during 96 weeks of follow-up among high-risk household contacts (HHCs) of adults with multidrug-resistant tuberculosis (MDR-TB)	clinicaltrials.gov : NCT03568383	3	Current	2019-2027 (estimated)	Excluded pregnant women but included lactating women	No, Excluded pregnant women but followed pregnancy outcomes for those who became pregnant on trial	National Institute of Allergy and Infectious Diseases (NIAID)
TB SCReening Improves Preventive Therapy Uptake-Moms (TB SCRIPT-Moms)		TB SCRIPT-Moms	2-stage pragmatic randomized trial to determine 1) the optimal TB screening strategy (symptom screen vs. C-reactive protein) to improve TB case detection and 2) the optimal short-course TPT delivery strategy (antenatal vs. postnatal) to improve maternal and birth outcomes among pregnant women with HIV in Uganda. This 2-stage pragmatic trial is proposed to take place in Kampala, Uganda, without restrictions on CD4 count, ART status or gestational age.	ClinicalTrials.gov: NCT04557176	3	current	2025-2030	Included	N/A	NHLBI
**TB therapeutic trials for TB prevention that excluded pregnant and/or breastfeeding women, but allowed them to stay on trial if they became pregnant (n=1)**										

A phase III cluster randomised placebo-controlled trial to assess the efficacy of preventive therapy in child and adolescent contacts of multidrug-resistant (MDR) tuberculosis (TB)		TB-CHAMP	TB-CHAMP will compare 24 weeks of daily levofloxacin (15-20 mg/kg, maximum 750 mg) against 24 weeks of daily placebo. All eligible children within the household will be treated with the same drug (either all levofloxacin or all placebo). Households will be randomised (allocated by chance) to be in either the levofloxacin or placebo group.	ISRCTN: ISRCTN92634082	3	Completed	2016-2024	Excluded at enrollment	YES, participants who became pregnant while on study were counselled and then given the option to either stop treatment or continue.	Stellenbosch University

Supplementary Table 2: Tuberculosis vaccine trials that excluded pregnant and/or lactating women at enrolment, but included them in in safety and/or immunogenicity assessments during follow-up, if they became pregnant on trial (n=6).

Vaccine Type	Official Study Name	Vaccine evaluated/Description of Trial	Registry ID	Trial Phase	Planned/ Current/ Completed	Year of Study	Included/Excluded Pregnant & Lactating Women at Enrollment	Allow Women who got pregnant after enrollment to remain during Trial follow-up? (Y/N)	Sponsor
Adjuvanted subunit	A Phase 3, Randomized, Double-blind, Placebo-controlled, Multicenter, Clinical Trial to Assess the Prophylactic Efficacy, Safety, and Immunogenicity of the Investigational M72/AS01E-4 Mycobacterium Tuberculosis (Mtb) Vaccine When Administered Intramuscularly on a 0,1-month Schedule to Adolescents and Adults	**M721/ASO1E-4**<br>The study is a randomized, double-blind, placebo-controlled, multicenter, clinical trial to assess the prophylactic efficacy, safety, and immunogenicity of the investigational M72/AS01E-4 Mtb vaccine when administered intramuscularly (IM) on a 0,1-month schedule to adolescents and adults. This trial will be conducted in 3 cohorts: Interferon gamma release assay (IGRA)-positive Cohort, IGRA-Negative Cohort and HIV Cohort.	clinicaltrials.gov : NCT06062238	3	Current	2024-2028	Excluded.<br>Female participants excluded with any one of the following conditions: currently pregnant or lactating; having positive serum pregnancy test during the screening window, positive urine pregnancy test on Day 1, planning a pregnancy within 2 months after completion of the vaccination series.	YES.<br>Maternal vaccine safety and some pregnancy outcomes captured. Immunogenicity evaluations were not performed among women who became pregnant.	Bill & Melinda Gates Medical Research Institute
Live whole cell	Efficacy of BCG Revaccination Compared With Oral Chemoprophylaxis in Household Contacts Aged 6-18 Years for Prevention of Tuberculosis Disease - A Phase III Open Labelled Randomised Controlled Trial	**BCG Re-vaccination**<br>The primary aim of this study is to assess the efficacy of BCG revaccination compared to oral chemoprophylaxis in preventing TB disease in house hold contacts aged 6-18 years. The study is designed as a multicentre randomised controlled trial with two groups of healthy household contacts aged 6-18 years receiving either the BCG vaccine or oral chemoprophylaxis. They will be followed up for 24 months to compare the incidence of TB disease in each arm.	clinicaltrials.gov : NCT05330884	3	Current	2024-2027	Excluded<br>Pregnancy was exclusion crtieria for female participants > 15 years of age will have pregnancy test done after caretakers and participants informed consent.	YES.<br>Maternal vaccine safety and some pregnancy outcomes captured. Immunogenicity evaluations were not performed among women who became pregnant.	Tuberculosis Research Centre, India
Live whole cell	Randomised, Double-Blind, Controlled Phase 3 Trial to Evaluate the Efficacy, Safety and Immunogenicity of MTBVAC in Healthy HIV Unexposed (HU) and HIV Exposed Uninfected (HEU) Newborns in Tuberculosis-Endemic Regions of Sub-Saharan Africa	**MTBVACN3**<br>The objective of this study is to demonstrate safety, immunogenicity and improved efficacy of the new live attenuated M. tuberculosis vaccine called MTBVAC in a Phase 3 efficacy trial in HIV-uninfected infants born to HIV-infected and HIV-uninfected mothers as compared to standard of care BCG vaccination.	clinicaltrials.gov : NCT04975178	3	Current	2022-2029	N/A - trial for newborns	YES.<br>Maternal vaccine safety and some pregnancy outcomes captured. Women who became pregnant were not exluded from immunogenicity evaluation	Biofabri, S.L
Live whole cell	A Multicenter Phase II/III Double-Blind, Randomized, Placebo Controlled Study To Evaluate The Efficacy And Safety Of VPM1002 In The Prevention Of TB Recurrence In Pulmonary TB Patients After Successful TB Treatment	**VPM1002**<br>This is a Phase II/III trial with two groups of adults successfully cured of category 1 pulmonary TB receiving either VPM1002 or placebo. Single dose of VPM1002 / placebo will be administered to calculate efficacy of the vaccine against TB recurrence.	clinicaltrials.gov : NCT03152903	2/3	Current	2017-2024	Excluded<br>Pregnant and / or lactating female participants, and must use reliable methods of birth control, have a negative pregnancy test during screening and have no intention to become pregnant for at least 3 months post-vaccination	YES.<br>Maternal vaccine safety and some pregnancy outcomes captured. Women who became pregnant were not exluded from immunogenicity evaluation	Serum Institute of India Pvt. Ltd.
Live whole cell	A Phase 2b, Double-blind, Randomized, Placebo-controlled Study to Evaluate the Efficacy, Safety and Immunogenicity of a Candidate TB Vaccine, MTBVAC, Against TB Disease in Interferon Gamma Release Assay Positive Adolescents and Adults Aged 14-45 Years, Living in a TB Endemic	**MTBVAC**<br>A Phase 2b, double-blind, randomized, placebo-controlled study to evaluate the efficacy, safety and immunogenicity of a candidate tuberculosis (TB) vaccine, MTBVAC, against TB disease in interferon gamma release assay positive adolescents and adults aged 14-45 years, living in a TB endemic region.	clinicaltrials.gov : NCT06272812	2b	Planned	2024-2028	Excluded	YES.<br>Maternal vaccine safety and some pregnancy outcomes captured. Women who became pregnant were not exluded from immunogenicity evaluation	International AIDS Vaccine Initiative
Adjuvanted subunit	A Phase 2a/2b Study Evaluating Safety, Immunogenicity, and Therapeutic Efficacy of ID93 + GLA-SE Vaccination in Participants With Rifampicin-Susceptible Pulmonary TB	**ID93 + GLA-SE**<br>This study is being done to test an experimental study vaccine compared to a placebo. The experimental study vaccine is called ID93 + GLA-SE. ID93 + GLA-SE has been used in humans in research but has not been approved for use in medical care. This study will be the first to test ID93 + GLA-SE in people living with HIV (PLWH). Injections will be given to different groups of participants while they are using standard TB treatment. One of the research questions is to understand the differences in immune system responses depending on the timing of giving the injections after people begin taking standard TB treatment. Researchers also want to continue to look at whether the study vaccine is safe when tested in a larger group of people, and if getting the study vaccine in addition to standard TB treatment can help to lower the number of poor TB outcomes that people might have.	clinicaltrials.gov : NCT06205589	2a/2b	Planned	2024-2029 (Not yet started, as of Jan 2026)	Excluded	YES.<br>Maternal vaccine safety and some pregnancy outcomes captured. Immunogenicity evaluations were not performed among women who became pregnant.	Advancing Clinical Therapeutics Globally for HIV/AIDS and Other Infections

Supplementary Table 3. Illustrative quotes for TB therapeutic trials per sub-theme for pregnancy and lactation

**Theme 1: Shifting the narrative**		
**Sub-themes**	**Illustrative quotes: Pregnancy**	**Illustrative quotes: Lactation**
TB trial design rooted in historic practices	"… it's a created practice… everybody knows you can't put a pregnant woman in the trials. … no IRB will approve that… We're not going to take that on; that's too much complexity.'" Y-10	"...Show me the compelling reason that we should not include lactating women. I'm not aware of what that would be." Y-10
Strict sponsor requirements	"… the sponsor requiring it... and the drugs being used not being approved for pregnancy. So of course, how do you get it approved for pregnancy if it's not allowed to be studied in pregnancy?" Y-02	
Clinical trial insurance	" … we had to have an insurance policy in each country… actually some insurance companies just refused... It was often very expensive… it's important to be aware…" L-03	"…it [concerns] will be regulatory, it will be PIs, it will be the pharmaceutical agents most of all. Yeah, and their concern will not only be safety, but it'll be monetary because of the increased costs of insurance." L-01
Lack of pragmatic guidelines	"... there weren't any clear pathways to involve pregnant women and children in these trials … we didn't know how to do it, and it was definitely easier to not do it." Y-04	
**Theme 2: Differentiated risks**		
**Sub-themes**	**Illustrative quotes: Pregnancy**	**Illustrative quotes: Lactation**

Separating pregnancy and lactation periods	"…in the end… if there is an exclusion for pregnancy, there will be also an exclusion for breastfeeding. If you can convince people to include pregnant women, then it will not be hard to convince them also to include breastfeeding women." L-03	"...we really didn't parse out pregnant from lactating …although now that we're thinking about it today, I can see that it's a different calculus." Y-04
Trimester	"…I think everybody's worried about first trimester studies just because the adverse event rate of pregnancy in the first trimester is so high without drugs. And you don't want to get a muddled signal on whether miscarriages have anything to do with a drug when you're not powered for that." Y-02	N/A
Drug Safety	"I think also it's really important to have specific PK data… the toxicities may be different for the woman but also for the… fetus so that it's really important that we get those data." L-02	"…understanding the drug levels and the transfer of the drug to the infant is very important. …, there's so much that's not known about the drug pharmacology and the lactation compartment..." S-03
Research context	"…for a lot of pregnant women, we also have to think about who's the decision maker… … So, we've even designed trials where… looking at that from a family-centered approach, even though you just want to include the woman." S-03	"…the breastfeeding practices are very, very different in different countries, …society's attitudes towards it, where people breastfeed, how they breastfeed, etc… those kinds of community pressures would substantially impact on whether a breastfeeding mother would feel comfortable engaging in a clinical trial." L-02
Weighing up risk-benefit	"…if it's therapeutics for treatment, the more severe the disease… you're willing to take more risk because the disease itself is associated with risk of a lot more harm, morbidity, mortality.." S-03	"…I don't think necessarily we know that if the amount of drug levels that gets into the breast milk is directly beneficial to the infant, but I think a healthy mom is a healthy baby." Y-05
**Theme 3: Perceived concerns of PLW**		

Sub-themes	Illustrative quotes: Pregnancy	Illustrative quotes: Lactation
Safety for the women	"…often the pregnant person is being told 'we don't have any data, this trial is being designed to give us data, this is all we know'. And so… there's a lot of pressure on a participant…" Y-03	"…when a patient who isn't given a heads up… and sees that it changed the colour of her breastmilk, of course she might worry about the safety… ? Y-08
Safety for the fetus/infant	"Well, obviously anything that a pregnant woman is exposed to has the potential of going to the fetus…?" Y-05	"…I think the concerns would be how much of these drugs get to my infant through breastmilk and are they safe? Y-03
Time Commitment	"… it's so time-consuming. And I can imagine a pregnant person… they've got other things in their life and they're pregnant and they may not be feeling well if they have active TB…" Y-03	"… around the time of delivery and postpartum, …[women] forget to take their medications because they're managing a new life and they have a lot going on at home. So sometimes that can be a problem" Y-05
**Theme 4: Strategies for inclusion of PLW in TB clinical trials**		
**Sub-themes**	**Illustrative quotes: Pregnancy**	**Illustrative quotes: Lactation**
Working closely with community	"… we know the power of community, especially a community where they feel they have been themselves impacted from TB and even more powerful if they've been impacted during pregnancy…" S-03	"But there has to be really a push …have people from the community inside the protocol writing group since the beginning, so there's no way they can forget. " L-03
Educating trial participants	"…knowledge is comfort… communicating as much as we know in the informed consent process … having them understand what… we do and don't know and therefore what the risks are is …the most important." Y-01	"I think education about the benefits of being on treatment at that critical time both for the mothers themselves and also their babies…." L-02

Sensitization within regulatory bodies	"I think that those groups [regulatory bodies] need to be educated and aware of the issues and of the movement in the field and of the reasons for doing this." Y-04	"…the regulatory bodies are going to fight tooth and nail saying 'no'… there's always a question of malformations or what was the side effect with the baby. They want to protect the baby and the mother." Y-06
Lactation is a good time for enrolling women in TB trials	N/A	"…I think that they [lactating women] should be offered [TPT trials] because… there's postpartum care that's going on. There's like early infant vaccine visits that are going on. So I think programmatically that might be a time to be able to provide these therapies…" Y-03

Supplementary Table 4. Illustrative quotes for vaccine trials per sub-theme for pregnancy and lactation

**Theme 1: Shifting the narrative**		
**Sub-themes**	**Illustrative quotes: Pregnancy**	**Illustrative quotes: Lactation**
TB trial design rooted in historic practices	"Easier to not take the risk. It's easier to not look at all the ethical issues and the what-ifs and how to go about it. But at some point, one's going to have to get over that." S-02	
Strict sponsor requirements	"… as a sponsor of a vaccine, you always have to think about, are you putting the product at risk unnecessarily in a way that might keep it from ever getting licensed for anybody and not be of benefit to anyone?" Y-11	

Lack of pragmatic guidelines		“I think somebody just has to be first. Like the hardest thing to do is be the first person who does it, but then after that, you set a standard of care.” Y-01
**Theme 2: Differentiated risks**		
**Sub-themes**	**Illustrative quotes: Pregnancy**	**Illustrative quotes: Lactation**
Separating pregnancy and lactation periods	“it's possible that your immune response to a vaccine could be somewhat suppressed during pregnancy. …see data on making sure that we get antibody levels that are at least comparable to what we saw in non-pregnant individuals.” Y-05	“…Lactation is a different time period from pregnancy because you don't have the same kind of immunological pressures that you do during pregnancy. But it would just be nice to know that this is a safe and efficacious thing to do.” S-01
Trimester	"…I think I would steer away from very early pregnancies. And the weird thing is that's probably when most of the vaccines will be given in error because the women don't know that they're pregnant. …probably the middle trimester would be the best..." S-01	N/A
Drug Safety	“I mean, ideally, it [vaccines] wouldn't affect the health of the fetus at all, I guess, except in a rare circumstance… I don't know that there is a circumstance where giving a vaccine has resulted in a teratogenic effect to the child…” Y-01	“I think really, I mean, you always have to think about the vaccine that you're giving and whatever safety is known and whatever animal studies have happened, like you should never just assume...” Y-01
Research context	“…also our laws are somewhat a barrier in the sense that if there is a pregnant woman, sometimes it requires both mother and father to consent. So, getting both of them on board may become a barrier.” Y-09	“There's a lot of women who go away, you know, and don't get care routinely in the postpartum period.” S-03

Weighing up risk-benefit	"…how do you handle the risk benefit in any vaccine trial? … We are making some assumptions based on immunogenicity and some preclinical data that they call protection data… And so if you don't know how the vaccines work, I mean if the vaccines will work. And if you don't even have a correlate …it becomes a hard decision about the nature of that risk to the pregnant woman, to the fetus." Y-07	"Different vaccine candidates may have different kind of risk weights. And there may be one candidate where there's a risk, but it's a lightweight risk. And there's another candidate where the risk is a heavier weight risk." Y-07
**Theme 3: Perceived concerns of PLW**		
**Sub-themes**	**Illustrative quotes: Pregnancy**	**Illustrative quotes: Lactation**
Safety for the women	"…I think also it would be somewhat vaccine dependent… Are you worried about an adverse event that could be triggered by the vaccine that wouldn't necessarily directly impact a pregnant woman but could indirectly impact a pregnant woman." Y-07	"…if the vaccine actually worked, preventing TB in a lactating woman could be hugely helpful to her, but also another factor that could prevent her infant from acquiring the TB infection." Y-07
Safety for the fetus/infant	"The main concern is … how it's going to affect the baby, whether the baby is going to be having, congenital anomalies, … born preterm, going to be small…." Y-06	"… first fundamental question is what's the risk-benefit for that individual? … for a lactating woman, we still don't know that there's not some component in that vaccine that might be harmful to the infant." Y-11
Time Commitment	"… How much time and energy is it going to take during this period where I'm not feeling great anyway…?" Y-11	
**Theme 4: Strategies for inclusion of PLW in TB clinical trials**		

Sub-themes	Illustrative quotes: Pregnancy	Illustrative quotes: Lactation
Working closely with community	"Well, they [community advocates] play a crucial role …it's important that we include them and use their power… So, I think the big thing for them is that they can pressurise the pharmaceuticals." L-01	"…if something goes wrong in pregnancy, it's going to be blamed on the vaccine and that's going to spread throughout the community… And so, the risks in pregnancy are slightly higher, so that's why I say it would be easier for the community to advocate for postpartum women." L-01
Educating trial participants	"…can help educate both the investigators, the trialists, but also the [pregnant] women, their neighbours and friends and relatives in the community as to what the pluses and minuses are …they really understand what risks they're taking as well as the potential benefit." Y-11	"…one is education. And two is that some women have already taken part before so their own experiences if they can be spoken by the women who took part in the vaccine trial, if she can talk to the patients who can, who are potential participants for the study, that would really help." Y-06
Sensitization within regulatory bodies	"… one will need to consult your local ethics committee… What would be acceptable to them? Because it's pointless planning an entire trial and your ethics committee throws it out …but I know there's more and more consultation and discussion amongst ethics committees to include pregnant women because otherwise it's crazy." S-02	"…you just have to protect [the vaccine] or else you're going to shut down vaccine development, or you just won't get vaccines developed..." Y-07
Lactation is a good time for enrolling women in TB trials	N/A	"If we find a new vaccine that is effective in adults, …very important as this is a time when I think women's immune system takes quite a knock from breastfeeding, It's very draining on a woman and this is perhaps when she's very vulnerable to TB and it would be a good time to give her new vaccine." S-02
